# The Inherited *KRAS*-variant as a Biomarker of Cetuximab Response in NSCLC

**DOI:** 10.1158/2767-9764.CRC-23-0084

**Published:** 2023-10-11

**Authors:** Joanne B. Weidhaas, Chen Hu, Ritsuko Komaki, Gregory A. Masters, George R. Blumenschein, Joe Y. Chang, Bo Lu, Adam P. Dicker, Jeffrey A. Bogart, Yolanda I. Garces, Samir Narayan, Clifford G. Robinson, Vivek S. Kavadi, Joel S. Greenberger, Christopher D. Koprowski, James Welsh, Elizabeth M. Gore, Robert M. MacRae, Rebecca Paulus, Jeffrey D. Bradley

**Affiliations:** 1 Department of Radiation Oncology, UCLA, Los Angeles, California.; 2 NRG Oncology Statistics and Data Management Center, Philadelphia, Pennsylvania.; 3 Sidney Kimmel Comprehensive Cancer Center, Johns Hopkins University School of Medicine, Baltimore, Maryland.; 4 MD Anderson Cancer Center, Houston, Texas.; 5 Helen F Graham Cancer Center and Research Institute and Medical Oncology Hematology Consultants Pa, Newark, Delaware.; 6 Thomas Jefferson University Hospital, Philadelphia, Pennsylvania.; 7 Upstate Medical University (accruals Thomas Jefferson University Hospital), Syracuse, New York.; 8 Mayo Clinic, Rochester, Minnesota.; 9 St. Joseph Mercy Cancer Center (accruals Michigan Cancer Research Consortium CCOP), Ypsilanti, Michigan.; 10 Washington University, St. Louis, Missouri.; 11 Texas Oncology Cancer Center Sugar Land, Sugar Land, Texas.; 12 UPMC-Shadyside Hospital, Pittsburgh, Pennsylvania.; 13 Helen F Graham Cancer Center (accruals Christiana Care Health Services, Inc. CCOP), Newark, Delaware.; 14 Medical College of Wisconsin and the Zablocki VAMC, Milwaukee, Wisconsin.; 15 The Ottawa Hospital, Ottawa, Ontario, Canada.; 16 Emory University School of Medicine, Atlanta, Georgia.

## Abstract

**Purpose:**

RTOG 0617 was a phase III randomized trial for patients with unresectable stage IIIA/IIIB non–small cell lung cancer comparing standard-dose (60 Gy) versus high-dose (74 Gy) radiotherapy and chemotherapy, plus or minus cetuximab. Although the study was negative, based on prior evidence that patients with the *KRAS-*variant, an inherited germline mutation, benefit from cetuximab, we evaluated *KRAS-*variant patients in RTOG 0617.

**Experimental Design:**

From RTOG 0617, 328 of 496 (66%) of patients were included in this analysis. For time-to-event outcomes, stratified log-rank tests and multivariable Cox regression models were used. For binary outcomes, Cochran—Mantel–Haenzel tests and multivariable logistic regression models were used. All statistical tests were two sided, and a *P* value <0.05 was considered significant.

**Results:**

A total of 17.1% (56/328) of patients had the *KRAS-*variant, and overall survival rates were similar between *KRAS-*variant and non-variant patients. However, there was a time-dependent effect of cetuximab seen only in *KRAS-*variant patients—while the hazard of death was higher in cetuximab-treated patients within year 1 [HR = 3.37, 95% confidence interval (CI): 1.13–10.10, *P* = 0.030], death was lower from year 1 to 4 (HR = 0.33, 95% CI: 0.11–0.97, *P* = 0.043). In contrast, in non-variant patients, the addition of cetuximab significantly increased local failure (HR = 1.59, 95% CI: 1.11–2.28, *P* = 0.012).

**Conclusions/Discussion:**

Although an overall survival advantage was not achieved in *KRAS-*variant patients, there is potential impact of cetuximab for this genetic subset of patients. In contrast, cetuximab seems to harm non-variant patients. These findings further support the importance of genetic patient selection in trials studying the addition of systemic agents to radiotherapy.

**Significance:**

The *KRAS-*variant is the first functional, inherited miRNA-disrupting variant identified in cancer. Our findings support that cetuximab has a potentially beneficial impact on *KRAS-*variant patients treated with radiation. The work confirms prior evidence that *KRAS-*variant patients are a subgroup who are especially sensitive to radiation. These findings further support the potential of this class of variants to enable true treatment personalization, considering the equally important endpoints of response and toxicity.

## Introduction

In the field of radiation oncology, there has been great interest in improving tumor local control, with the addition of radiosensitizing agents as well as with dose escalation. Non–small cell lung cancer (NSCLC) is a cancer type with many such efforts, as local failure (LF) remains unacceptably high, especially in patients with advanced stage disease (1). RTOG 0617 was designed on the basis of encouraging data from a phase II trial in NSCLC (RTOG 0324), where combined chemoradiation with the radiosensitizing agent, cetuximab, in patients with unresectable stage III NSCLC showed a median survival of 22.7 months with a 24-month overall survival (OS) of 49.3% (2). In addition, phase I and II trials showed that a tumor dose of 74 Gy given with chemotherapy appeared to be safe and achieved a median OS of approximately 24 months (3, 4). Therefore, RTOG 0617 was built upon these findings as an open-label, randomized, two-by-two factorial phase III study, which was conducted in 185 institutions in the United States and Canada. In this study, 544 patients with stage III NSCLC were enrolled onto one of four arms, 60 Gy (*n* = 166), 74 Gy (*n* = 121), 60 Gy plus cetuximab (*n* = 147), or 74 Gy plus cetuximab (*n* = 110). Unfortunately, results from this trial were negative, with the highest median survival of 28.7 months achieved in the standard-dose arm without cetuximab. Both the radiation dose escalation and cetuximab results crossed futility boundaries and there was evidence that the dose escalation was harmful to patients, likely due to increased heart dose (3, 5), and that the use of cetuximab was also associated with significantly higher toxicity. Additional efforts to try to identify potential cetuximab responders in RTOG 0617 included evaluation of EGFR expression, measured as the H-score, but also found no association with benefit (3).

The negative results from RTOG 0617 were not dissimilar from several other phase III trials combining cetuximab with radiotherapy, including one in esophageal cancer (RTOG 0436; ref. 6), and one in head and neck squamous cell carcinoma (HNSCC; RTOG 0522; ref. 7). In addition, two follow-up phase III studies in HNSCC that were designed to test the hypothesis that cetuximab with radiation would be a less toxic and equivalent option compared with cisplatin and radiation for human patients with papillomavirus–positive (HPV+) HNSCC were both negative. In fact, both trials reported worse outcomes in the cetuximab-treated arms, with worse 2-year OS and 2-year recurrence in low-risk HPV+ oropharyngeal cancer [De-ESCALaTE HPV, (8)], and worse progression-free survival (PFS) and locoregional failure [LRF] for all-risk HPV+ oropharyngeal cancer [RTOG 1016, (9)]. The litany of failed phase III trials incorporating cetuximab in combination with radiation after positive phase II trials suggests that there may be subgroups of patients who benefit from cetuximab, but that they have not been appropriately represented in subsequent phase III trials.

Recently, a germline, inherited variant in *KRAS,* referred to as the *KRAS-*variant, was identified as the first example of an inherited miRNA binding-site pathogenic variant in cancer (10). This inherited *KRAS-*variant is not a protein coding difference found in patients’ tumors, but instead is germline and in all cells and located in the regulatory 3′ untranslated region of *KRAS*. The *KRAS-*variant has not been found to be associated with tumor acquired *KRAS* mutations, and while not mutually exclusive, is found less frequently in patients with NSCLC who have tumor acquired *EGFR* or *KRAS* mutations (4% and 11%, respectively, vs. ∼17%–20%, (10, 11)]. However, even in the absence of tumor acquired *KRAS* mutations, patients with the *KRAS-*variant have a “*KRAS*-addicted” gene expression signature in their respective tumors (12), perhaps due to disruption of *KRAS* regulation by the miRNA *let-7*. Because of the *KRAS-*variants’ association with *KRAS,* there has been extensive study of the impact of cetuximab on patients with cancer with the *KRAS-*variant. Interestingly, in contrast to tumor acquired *KRAS* mutations which predict cetuximab resistance, patients with the *KRAS-*variant have repeatedly been shown to respond favorably to cetuximab alone or in combination with chemotherapy (13, 14).

Because of the benefit of cetuximab for *KRAS-*variant patients, the *KRAS-*variant was previously evaluated in the RTOG 0522 HNSCC trial, to determine the impact of cetuximab in combination with radiation and chemotherapy for *KRAS-*variant patients. The *KRAS-*variant was found in approximately 16% of RTOG 0522 patients, and this molecular subgroup was found to benefit significantly from the addition of cetuximab to radiotherapy and cisplatin chemotherapy, resulting in improved PFS and OS in a time-dependent manner (15). The time-dependent benefit of cetuximab resulted in improved survival for the first 2 years (*P* = 0.03), the short nature of which was hypothesized to potentially be due to the short course of cetuximab in the study (8 weeks). The positive response was found to be strongest in the HPV+ patients, and hypothesized to be due to the potential immune stimulating effects of cetuximab (16–18), as this study also demonstrated that *KRAS-*variant patients had significantly elevated TGF-B1 compared with non-variant patients, and thus *KRAS-*variant patients were likely immune suppressed (15). In addition, perhaps because of their elevated TGF-B1, *KRAS-*variant patients were found to have increased baseline acute grade 3 and 4 toxicity with radiation plus cisplatin in this study, that was not increased by the addition of cetuximab; including mucositis (47.4% vs. 50.0%) and skin reaction inside the portal (18.4% vs. 15.6%). This was in contrast to non-variant patients, who had lower toxicity with radiation and cisplatin which was significantly increased with the addition of cetuximab; including mucositis (37.9% vs. 50.6%, *P* = 0.02) and skin reaction inside the portal (11.2% vs. 21.8%, *P* = 0.05). Both *KRAS-*variant and non-variant patients had significantly increased skin reactions outside of the portal with the addition of cetuximab, which was not correlated with cetuximab response in that trial.

On the basis of these prior findings, that *KRAS-*variant patients respond to cetuximab in combination with radiation and cisplatin with improved outcomes, but with increased radiosensitivity at baseline (15), and prior work indicating that the *KRAS-*variant is found frequently in patients with NSCLC (10), we tested patients from RTOG 0617 for the *KRAS-*variant to investigate the outcomes for this genetic subgroup of patients.

## Materials and Methods

### Patients, DNA Isolation, and *KRAS*-variant Testing

All patients included in this study were consented for the clinical trial RTOG 0617, which was conducted in accordance with recognized ethical guidelines. All patient samples were received from the RTOG/NRG sample repository as anonymized blood or buffy coat samples. All DNA was isolated using Qiagen standard protocols, evaluated for purity, and quantified and prepared for *KRAS-*variant analysis by MiraDx using standard operating procedures. Patients are considered positive for the *KRAS-*variant if they are heterozygous (GT) or homozygous (GG) for the variant allele.

### Statistical Analyses

This analysis was performed using data from all eligible patients from RTOG 0617. Eligible patients who did not receive radiation or received ≤51 Gy were excluded from this analysis. All analyses were based on “as-treated” populations for this secondary analysis. Specifically, patients were considered as receiving a “standard” dose of radiation if they received more than 51 Gy but less than or equal to 66 Gy and receiving a “high” dose of radiation if they received more than 66 Gy; patients were considered as receiving cetuximab if they received cetuximab during concurrent therapy or beyond, and receiving no cetuximab if they did not receive any cetuximab or received only the loading dose.

The efficacy outcomes included in this analysis included OS, local failure (LF), distant failure (DF), and PFS, as defined in Supplementary Table S1. Adverse events were assessed using the CTCAE v3.0.

The prognostic value of the *KRAS-*variant was evaluated by (i) comparing *KRAS*-variant and non-variant patients after stratifying by radiation dose and cetuximab; (ii) multivariable regression model, including *KRAS*-variant, radiation dose, as well as their interaction, after stratifying by cetuximab; (iii) multivariable regression model, including *KRAS*-variant, cetuximab, as well as their interaction, after stratifying by radiation dose. In addition, the predictive value of the *KRAS*-variant was evaluated through subgroup analysis within *KRAS-*variant and non-variant patients, by comparing radiation dose groups (stratified by cetuximab) or cetuximab groups (stratified by radiation doses). These analysis methods were used for all clinical outcomes studied. For time-to-event outcomes, stratified log-rank tests and multivariable Cox regression models were used. For binary outcomes, Cochran–Mantel–Haenzel tests and multivariable logistic regression models were used.

OS, PFS, time to LF, and time to DF were analyzed as time-to-event data and calculated from the date of randomization to the date of failure or, if no failure, the date of respective competing event or last follow-up. The rates of OS and PFS were estimated using the Kaplan–Meier method and compared using the stratified log-rank test (19). The rates of LF were estimated using cumulative incidence function (19) which takes into account competing risks (see Supplementary Table S1 for details). The development of LF and distant metastasis were compared using the cause-specific competing risks analysis method (20), and the corresponding stratified log-rank test *P* values were reported accordingly. Multivariable Cox regression model was used when evaluating the prognostic value of *KRAS*-variant.

Best observed response was analyzed as a binary outcome, whether complete or partial response was achieved versus otherwise. Safety was analyzed as a binary outcome, whether worst treatment-related toxicity was grade 3 or higher versus otherwise. Cochran–Mantel–Haenzel tests and multivariable logistic regression model were used to analyze these two endpoints.

All statistical tests were two sided, and a *P* value <0.05 was considered statistically significant. SAS v9.4 (SAS Institute) was used for all statistical analyses. Representativeness of the study participants are shown in Supplementary Table S2.

### Data Availability

The data on *KRAS-*variant genotyping in this study are available on request from the corresponding author.

## Results

### Comparison of Patients Included Versus Excluded

As only a subset of patients had samples available for *KRAS*-variant genotyping and/or completed treatment, we first evaluated whether patients who were included in the analysis were systematically different from those who were not*.*Supplementary Table S3 summarizes the patients’ inclusion status and Supplementary Table S4 summarizes and compares pretreatment characteristics based on analysis inclusion (vs. exclusion). These pretreatment characteristics were chosen based on the primary reporting and published secondary analyses. We found that patients included in this *KRAS* analysis appeared to receive 3D-conformal radiotherapy (CRT) versus intensity-modulated radiotherapy (IMRT) more frequently than those excluded (*P* = 0.0006). In addition, as all patients included in the analysis received at least 51 Gy, this was significantly different from the excluded patients and cohort overall, where 14.9% and 5% did not receive this amount of radiation, respectively (*P* < 0.0001). Otherwise, patients included or excluded were similar.

Patients included in this analysis had a median follow-up of 2.03 years, and 5.13 years among surviving patients, which did not differ based on analysis inclusion status. Furthermore, patients included in this analysis were not statistically different from those excluded in terms of OS (median survival 2.1 vs. 1.8 years, *P* = 0.81)), PFS (median survival 0.9 vs. 0.8 years, *P* = 0.59), LF (5-year rate 44.3% vs. 36.2%, *P* = 0.10) or distant metastasis (DM) (5-year rate 56.0% vs. 51.8%, *P* = 0.43). However, patients included tended to have slightly better observed responses with more complete/partial responses (62.2% vs. 52.4%, *P* = 0.04; Supplementary Table S5). They also had more grade 3+ treatment-related toxicity (82.3% vs. 70.8%, *P* = 0.001; Supplementary Table S6). These results suggest patients included in this analysis may be roughly considered as a random sample of the full RTOG 0617 patient population, although some caution should be taken when extrapolating the results of best observed response and toxicity for this group.

### *KRAS*-variant Versus Non-variant Patients

In this study, 17.1% (56/328) of included patients had the *KRAS-*variant. Comparing pretreatment characteristics of patients with the *KRAS-*variant versus without the *KRAS-*variant (non-variant) there were no significant differences, except there were more PET-staged non-variant patients than *KRAS*-variant patients (91.2% vs. 82.1%, *P* = 0.04; Supplementary Table S7). There were similar proportions of patients with the *KRAS-*variant across different NSCLC subtypes, including squamous versus non-squamous (*P* = 0.21). There were also similar stage groupings between *KRAS-*variant and non-variant patients. These findings are consistent with prior findings for patients with *KRAS-*variant NSCLC (10).

In addition, treatment was similar between *KRAS-*variant and non-variant patients, considering as assigned and as treated (Supplementary Table S7). For non-variant as well as for *KRAS-*variant patients, comparing the standard-dose versus high-dose arms, there were no significant differences considering age, gender, ethnicity, performance status, radiation technique, PET staging, histology, stage, tumor location, radiation level, or cetuximab treatment. There were also no significant differences in these factors for non-variant and variant patients treated with cetuximab, versus not.

### OS, Radiation Dose, and Cetuximab in Non-variant versus KRAS-variant Patients

Overall, there was not a significant difference in OS between non-variant and *KRAS-*variant patients (2.1 vs. 2.4 years, *P* = 0.42; Supplementary Table S8). However, because dose escalation has now been identified as an independent risk for death in this trial, and *KRAS-*variant patients have been shown to have increased toxicity with radiation and chemotherapy at baseline, as well as a time-dependent effect of cetuximab (15), we further evaluated the impact of response considering these variables.

**FIGURE 1:**
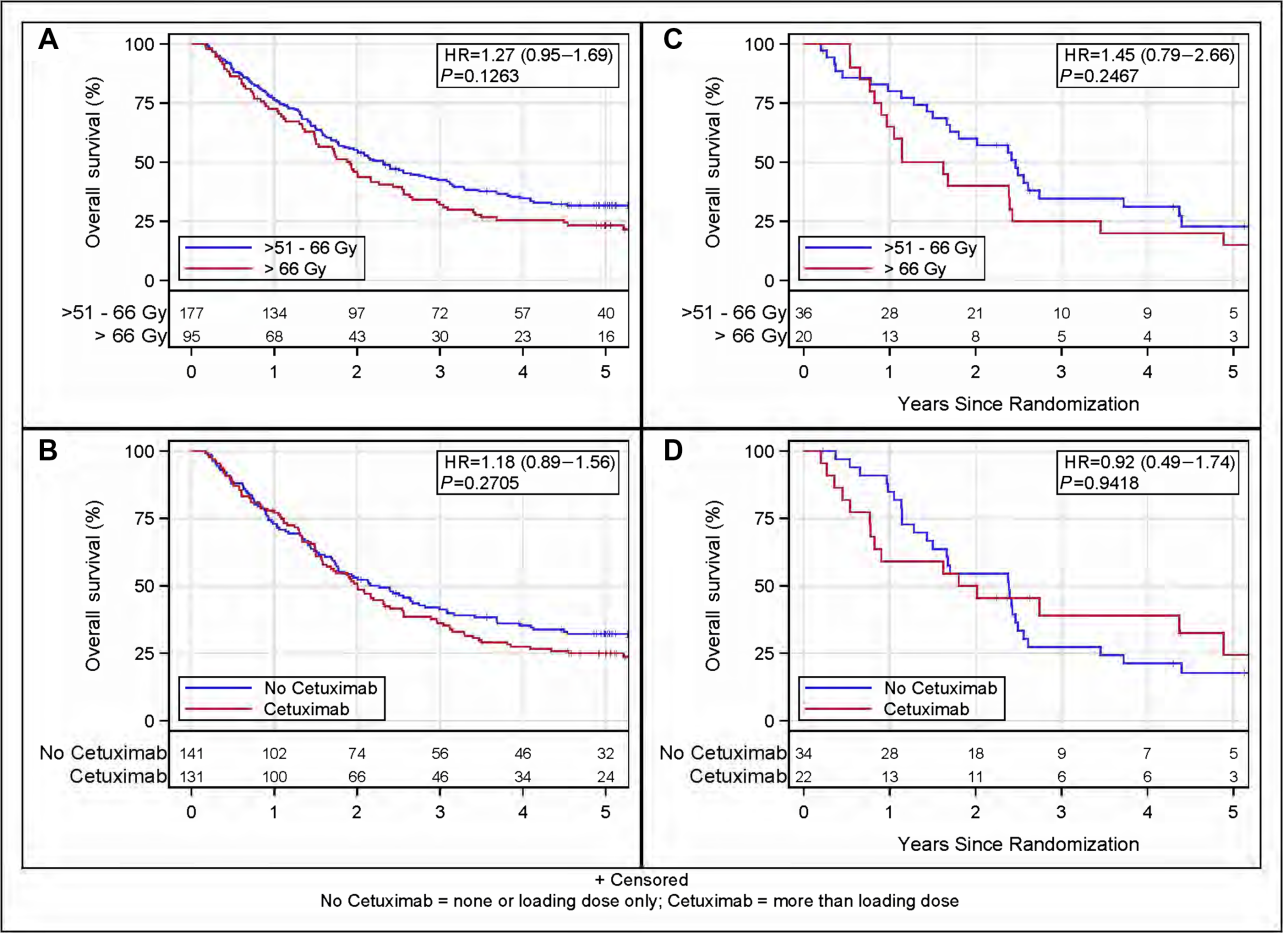
OS by *KRAS* and treatment arm. **A,** OS in non-variant patients by radiation level; **B,** OS in non-variant patients by cetuximab; **C,** OS in *KRAS*-variant patients by radiation level; **D,** OS within *KRAS*-variant patients by cetuximab. Radiation level *P* values stratified by cetuximab; cetuximab *P* values stratified by radiation level.

For non-variant and *KRAS-*variant patients, high-dose radiation appeared to nonsignificantly negatively impact survival over the 5-year follow-up period. Patients on the standard-dose arm had a trend for a longer median survival than those treated in the high-dose arm (non-variant 2.3 vs. 1.9 years, *P* = 0.13, Fig. 1A; *KRAS-*variant 2.5 vs. 1.4, *P* = 0.2467, Fig. 1C; Supplementary Table S9). For *KRAS-*variant patients, there was better survival during the first year for the standard-dose radiation group versus the high-dose radiation group, resulting in more *KRAS-*variant patients who received standard-dose radiation alive at 1 year (80.1% vs. 65.0%). These findings suggest that the high-dose treatment negatively affected patients regardless of *KRAS-*variant status, but that the negative impact may have been especially impactful for *KRAS-*variant patients.

We next evaluated the effect of cetuximab on OS, considering *KRAS-*variant status and time. In RTOG 0617, cetuximab was delivered for 15 or 16 weeks. In non-variant patients, there was no difference in OS or time-dependent OS between the cetuximab versus no cetuximab-treated group, but the cetuximab-treated group had a numerically nonsignificant shorter median survival (2.0 vs. 2.3 years *P* = 0.27; Supplementary Table S10; Fig. 1B). *KRAS-*variant patients also had a numerically nonsignificant shorter median survival when receiving cetuximab versus not (1.9 vs. 2.4 years, *P* = 0.94). However, unlike non-variant patients, in *KRAS-*variant patients treated with cetuximab the worse outcome seemed to occur primarily in year 1, with fewer *KRAS-*variant patients treated with versus without cetuximab alive at the end of year 1 (59.1% vs. 84.8%; Table 1). However, in contrast, there were almost twice as many *KRAS-*variant cetuximab-treated patients alive at the end of year 4 compared with non–cetuximab-treated patients (39.0% vs. 21.2%). We found that for *KRAS-*variant patients there was a time-dependent effect of cetuximab on OS; while the hazard of death was significantly greater in year 1 for *KRAS-*variant cetuximab-treated patients (HR = 3.37, 95% CI: 1.13–10.1, *P* = 0.030), the risk of death was significantly lower for *KRAS-*variant cetuximab-treated patients from years 1 to 4 (HR = 0.33, 95% CI: 0.11–0.97, *P* = 0.043; Table 2; Fig. 1D). These findings suggest that cetuximab appeared to be beneficial for *KRAS-*variant patients who survived the first year of treatment.

**TABLE 1:** OS within *KRAS*-variant patients by as-treated cetuximab

	No Cetuximab		Cetuximab	
Time (years)	% Alive (95% CI)	No. of patients at risk	% Alive (95% CI)	No. of patients at risk
0	100% (N/A)	34	100% (N/A)	22
1	84.8% (67.4–93.4)	28	59.1% (36.1–76.2)	13
2	54.5% (36.3–69.6)	18	50.0% (28.2–68.4)	11
3	27.3% (13.6–42.9)	9	39.0% (18.5–59.1)	6
4	21.2% (9.4–36.3)	7	39.0% (18.5–59.1)	6
5	17.7% (6.9–32.5)	5	24.4% (7.4–46.5)	3
Dead/Total	29/34		15/22	
Median survival time (95% CI)	2.4 (1.4–2.5)		1.9 (0.8–4.9)	
HR (95% CI)	0.92 (0.49–1.74)			
*P* ^a^	0.9418			

^a^Two-sided log-rank, stratified by radiation level (>51 Gy–≤66 Gy vs. >66 Gy).

**TABLE 2:** OS models within *KRAS*-variant: time-varying cetuximab effect

Time-varying model set	Variable	Comparison	HR (95% CI)	*P*
One year only	Cetuximab within 1 year	No Cetuximab (RL) vs. Cetuximab	3.37 (1.13–10.1)	0.0298
	Cetuximab >1 year	No Cetuximab (RL) vs. Cetuximab	0.45 (0.18–1.10)	0.0793
	Radiation level	>51 Gy–≤66 Gy vs. >66 Gy	1.43 (0.78–2.63)	0.2434
Years 1 and 2	Cetuximab within 1 year	No Cetuximab (RL) vs. Cetuximab	3.37 (1.13–10.1)	0.0299
	Cetuximab from year 1 to year 2	No Cetuximab (RL) vs. Cetuximab	0.37 (0.08–1.71)	0.2039
	Cetuximab >2 years	No Cetuximab (RL) vs. Cetuximab	0.49 (0.16–1.53)	0.2224
	Radiation level	>51 Gy–≤66 Gy vs. >66 Gy	1.43 (0.78–2.62)	0.2479
Years 1 and 4	Cetuximab within 1 year	No Cetuximab (RL) vs. Cetuximab	3.37 (1.13–10.1)	0.0299
	Cetuximab from year 1 to year 4	No Cetuximab (RL) vs. Cetuximab	0.33 (0.11–0.97)	0.0429
	Cetuximab >4 years	No Cetuximab (RL) vs. Cetuximab	1.66 (0.21–12.9)	0.6265
	Radiation level	Standard dose (RL) vs. High dose	1.42 (0.78–2.61)	0.2527

### PFS, Local Control, and Distant Metastases-free Survival

There were no significant PFS differences between non-variant and *KRAS-*variant patients for the whole group, or considering dose groups, or cetuximab treatment. The *KRAS-*variant was also not prognostic or predictive for LF or DF risk considering all patients compared with non-variant patients.

Interestingly however, in non-variant patients, cetuximab treatment predicted a significantly higher LF risk than no cetuximab treatment [HR = 1.59 (CI = 1.11–2.28, log-rank test *P* = 0.01)] (Table 3). In contrast, cetuximab was not associated with an increased LF risk in *KRAS-*variant patients (Supplementary Table S11).

**TABLE 3:** LF within non-variant patients by as-treated cetuximab

	No Cetuximab		Cetuximab	
Time (years)	% Alive (95% CI)	No. of patients at risk	% Alive (95% CI)	No. of patients at risk
0	0% (N/A)	141	0% (N/A)	131
1	14.9% (9.6–21.4)	87	27.5% (20.1–35.4)	73
2	26.4% (19.4–33.9)	59	46.1% (37.3–54.4)	39
3	35.1% (27.2–43.0)	40	48.4% (39.5–56.8)	30
4	37.3% (29.2–45.3)	33	50.0% (41.1–58.3)	24
5	38.1% (30.0–46.1)	25	50.8% (41.9–59.1)	17
Dead/Total	53/141		67/131	
HR (95% CI)	1.59 (1.11–2.28)			
*P* ^a^	0.0120			

NOTE: Model stratified by radiation level assignment.

^a^Two-sided log-rank, stratified by radiation level (>51 Gy–≤66 Gy vs. >66 Gy).

### Toxicity

We next evaluated the association of the *KRAS*-variant with grade 3+ treatment-related toxicity. Overall, patients with the *KRAS*-variant had similar toxicity rates as non-variant patients, with no differences comparing hematologic toxicity, pulmonary toxicity, or esophagitis after adjusting for (stratifying by) radiation dose and cetuximab [Supplementary Table S12, 83.9% vs. 83.1%, OR = 1.18 (CI: 0.53–2.60), *P* = 0.69]. Comparing the high-dose versus the standard radiation dose treatment arms, dose was not found to be a significant predictor of increased toxicity using logistic regression models (Supplementary Table S13). However, while there was no apparent difference in grade 4 toxicity in non-variant patients between the high-dose and standard-dose arms (34.7% vs. 32.2%), the difference in grade 4 toxicity appeared to be greater for *KRAS-*variant patients in the high-dose arm versus the standard-dose arm (40.0% vs. 22.2%).

Cetuximab, in contrast, was an independent predictor for significantly higher toxicity for both non-variant and *KRAS-*variant patients (Table 4, OR = 3.42, CI: 1.76–6.67, *P* = 0.0003), but was not specific to hematologic, pulmonary, or esophagitis. Both non-variant and *KRAS*-variant patients had significantly increased toxicity when cetuximab was delivered with treatment (within non-variant, *P* = 0.003; within *KRAS-*variant *P* = 0.008; Supplementary Tables S14 and S15). For *KRAS-*variant patients, all had grade 2 or higher toxicity, and 100% of those treated with cetuximab had grade 3 or higher toxicity, with 13.6% of cetuximab-treated *KRAS-*variant patients experiencing grade 5 events. Of note, it was not possible to evaluate acute versus late toxicity separately in this analysis.

**TABLE 4:** Worst treatment-related toxicity logistic regression model of KRAS and cetuximab interaction

Toxicity	Variable	Comparison	OR (95% CI)	p-value
Any grade 3+ Toxicity	KRAS mutation type	Non-variant (RL) vs. Variant	1.17 (0.53–2.60)	0.69
	Cetuximab	No Cetuximab (RL) vs. Cetuximab	3.42 (1.76–6.67)	0.0003

### *KRAS*-variant Squamous Patients

Because cetuximab has been generally used as a radiosensitizer for squamous cell cancers, and the prior benefit of cetuximab for patients with *KRAS-*variant cancer in combination with radiation was in squamous cell HNSCC, we compared *KRAS-*variant with non-variant patients with squamous histology to evaluate their outcome in this cohort. We found that patients with *KRAS-*variant squamous cell lung cancer had significantly worse OS than non-variant squamous patients (HR = 1.76, CI = 1.07–2.90, log-rank test *P* = 0.0307 stratified by as-treated radiation dose level and cetuximab). This difference appeared to be driven primarily by DF, with a significantly higher rate of DF in *KRAS-*variant versus non-variant patients with squamous NSCLC (HR = 1.68, CI = 0.90, 3.15, log-rank test *P* = 0.0362 stratified by as-treated radiation level and cetuximab). Although due to the small sample size, it was not possible to meaningfully evaluate the impact of cetuximab on this subgroup, cetuximab did appear to improve OS for *KRAS-*variant patients comparing survival at year 1 for cetuximab-treated versus non–cetuximab-treated patients (92.3% vs. 37.5%), but the overall impact was not significant (HR = 0.94, CI = 0.35, 2.54, log-rank test *P* = 0.95; Supplementary Table S16).

## Discussion

In this phase III trial of radiation dose escalation in locally advanced NSCLC with or without cetuximab, we performed a hypothesis-driven subgroup analysis to determine whether inherited *KRAS-*variant patients with NSCLC benefitted from cetuximab. Although there were a relatively small number of *KRAS-*variant patients in each arm due to the multiple interventions in this trial, and we did not see an OS advantage for *KRAS-*variant cetuximab-treated patients, there were some interesting findings in this analysis. First, there was what appeared to be a time-dependent benefit of cetuximab for *KRAS-*variant patients with NSCLC, who exhibited significantly improved OS from year 1 to 4 when they received cetuximab. However, *KRAS-*variant patients appeared to have toxicity and do poorly with treatment intensification, including radiation dose escalation, or cetuximab therapy, with poor survival in the first year of treatment across groups. In addition, and in agreement with findings in other trials combining radiation and cetuximab, non-variant patients had significantly worse outcomes with the addition of cetuximab, with worse local control, indicating that cetuximab may be especially harmful to non-variant patients.

The time-dependent effects of cetuximab seen in this trial are similar to the findings in RTOG 0522, where patients with HNSCC were treated with 8 weeks of cetuximab and the subgroup of *KRAS-*variant patients had an OS benefit that lasted for 2 years. In RTOG 0617, cetuximab was given for approximately twice as long, or 16 weeks, and the OS benefit, after year 1, lasted for 4 years. One could hypothesize that the longer cetuximab treatment in 0617 extended the survival benefit for *KRAS-*variant patients. It is also interesting that in 0617 patients with squamous cell NSCLC with the *KRAS-*variant appeared to have significantly worse outcomes than non-variant squamous patients, with a potential benefit of cetuximab. This might better represent the population treated in 0522 and may be the group with the greatest benefit from the combination of cetuximab and radiation. Unfortunately, this subgroup in 0617 was too small for proper analysis of cetuximab benefit in this study.

Another important finding from RTOG 0522 further validated in this study is that *KRAS-*variant patients appear to be at risk of significant toxicity from both dose-escalated radiation as well as cetuximab. In 0617, these sensitivities may have led to their early death, explaining the poor survival in *KRAS-*variant patients in year 1 in both the dose-escalation group as well as the cetuximab-treated group. Although excessive death was not seen in *KRAS-*variant patients treated in 0522, the tissues treated in 0522 differ from 0617, with the heart as the suspected tissue at risk for the high toxicity and death and poor survival seen in 0617. The ability to truly differentiate death due to treatment versus death due to cancer remains a true challenge in clinical trials, especially those in difficult to control cancers where aggressive treatment is necessary to try to improve survival. NSCLC is one such cancer, and has the additional risk associated with treatment near to organs that are sensitive to radiation yet critical for survival.

We do not yet fully understand the mechanisms leading to radiosensitivity in *KRAS-*variant patients, nor do we fully understand the cause of increased radiosensitivity with the delivery of cetuximab, even after using this treatment in combination with radiation in numerous clinical trials. One simple explanation for radiosensitization in both *KRAS-*variant patients and with cetuximab may be that disruption of normal signaling through the *EGFR-KRAS* axis, as is found in patients with the *KRAS-*variant (12) and with cetuximab treatment, impairs a normal radiation response (21). However, the radiosensitivity in *KRAS-*variant patients could also be due to elevated TGF-B1 as reported previously (15) or perhaps due to altered *let-7* levels found in these patients (10, 12), as *let-7* is known to also known to be a critical player in the radiation response (22). The importance of better understanding causes of radiosensitivity as well as finding additional biomarkers of individual radiosensitivity cannot be overstated, as without this information, as a field we are significantly limited in advancing cure rates through combination therapies, if patients who may benefit from combination therapies die before exhibiting the benefit. This is work that is ongoing.

Although the results from this study are unlikely to lead to changes to improve outcome for patients with NSCLC, there are important takeaways from this analysis, and the findings offer some hope for the future. The miRNA-based germline pathogenic variants of which the inherited *KRAS-*variant is the first example, are a compelling class of biomarkers that may ultimately help us dramatically improve our approach to cancer therapy. Through their ability to identify patients with altered response to cancer treatment, as well as altered toxicity to cancer treatment, they could enable a future where patient treatment selection is genetically based, and that in addition to cancer control, treatment decisions will include the equally important endpoint of patient toxicity.

## Supplementary Material

Supplementary Data Table 1 Endpoints evaluated in RTOG 0617

Supplementary Data Table 2 Representativeness of Study Participants

Supplementary Data Table 3 KRAS Analysis Inclusion Status

Supplementary Data Table 4 Pretreatment Characteristics by KRAS Analysis Inclusion Status

Supplementary Data Table 5 Best Observed Response by KRAS Analysis Inclusion Status

Supplementary Data Table 6 Worst Overall Treatment-Related Adverse Event by KRAS Analysis Inclusion Status

Supplementary Data Table 7 Pretreatment Characteristics by KRAS-variant or non-variant status

Supplementary Data Table 8 Overall Survival

Supplementary Data Table 9 Overall Survival for non-variant and KRAS-Variant Patients by RT Level Assignment

Supplementary Data Table 10 Overall Survival within non-variant Patients Cetuximab Assignment

Supplementary Data Table 11 Local Failure within KRAS Variant Patients by As-Treated Cetuximab

Supplementary Data Table 12 Worst Treatment-Related Toxicity By KRAS Genotype

Supplementary Data Table 13 Worst Treatment-Related Toxicity Logistic Regression Model of KRAS and As-Treated RT Interaction

Supplementary Data Table 14 Worst Treatment-Related Toxicity within Non-variant Patients By Cetuximab Assignment

Supplementary Data Table 15 Worst Treatment-Related Toxicity within KRAS-Variant Patients By Cetuximab

Supplementary Data Table 16 Overall Survival Squamous Cell Variant Patients Only

## References

[1] Rajpara RS , Schreibmann E, Fox T, Stapleford LJ, Beitler JJ, Curran WJ, . Locoregional tumor failure after definitive radiation for patients with stage III non-small cell lung cancer. Radiation Oncol 2014;9:187. 10.1186/1748-717X-9-187 PMC4155086 25154893

[2] Blumenschein GR Jr , Paulus R, Curran WJ, Robert F, Fossella F, Werner-Wasik M, . Phase II study of cetuximab in combination with chemoradiation in patients with stage IIIA/B non-small-cell lung cancer: RTOG 0324. J Clin Oncol 2011;29:2312–8. 21555682 10.1200/JCO.2010.31.7875 PMC3107747

[3] Bradley JD , Paulus R, Komaki R, Masters G, Blumenschein G, Schild S, . Standard-dose versus high-dose conformal radiotherapy with concurrent and consolidation carboplatin plus paclitaxel with or without cetuximab for patients with stage IIIA or IIIB non-small-cell lung cancer (RTOG 0617): a randomised, two-by-two factorial phase 3 study. Lancet Oncol 2015;16:187–99. 25601342 10.1016/S1470-2045(14)71207-0 PMC4419359

[4] Socinski MA , Blackstock AW, Bogart JA, Wang X, Munley M, Rosenman J, . Randomized phase II trial of induction chemotherapy followed by concurrent chemotherapy and dose-escalated thoracic conformal radiotherapy (74 Gy) in stage III non-small-cell lung cancer: CALGB 30105. J Clin Oncol 2008;26:2457–63. 18487565 10.1200/JCO.2007.14.7371

[5] Speirs CK , DeWees TA, Rehman S, Molotievschi A, Velez MA, Mullen D, . Heart dose is an independent dosimetric predictor of overall survival in locally advanced non-small cell lung cancer. J Thorac Oncol 2017;12:293–301. 27743888 10.1016/j.jtho.2016.09.134

[6] Suntharalingam M , Winter K, Ilson D, Dicker AP, Kachnic L, Konski A, . Effect of the addition of cetuximab to paclitaxel, cisplatin, and radiation therapy for patients with esophageal cancer: the NRG Oncology RTOG 0436 phase 3 randomized clinical trial. JAMA Oncol 2017;3:1520–8. 28687830 10.1001/jamaoncol.2017.1598 PMC5710193

[7] Machtay M , Moughan J, Trotti A, Garden AS, Weber RS, Cooper JS, . Factors associated with severe late toxicity after concurrent chemoradiation for locally advanced head and neck cancer: an RTOG analysis. J Clin Oncol 2008;26:3582–9. 18559875 10.1200/JCO.2007.14.8841 PMC4911537

[8] Mehanna H , Robinson M, Hartley A, Kong A, Foran B, Fulton-Lieuw T, . Radiotherapy plus cisplatin or cetuximab in low-risk human papillomavirus-positive oropharyngeal cancer (De-ESCALaTE HPV): an open-label randomised controlled phase 3 trial. Lancet 2019;393:51–60. 30449623 10.1016/S0140-6736(18)32752-1 PMC6319250

[9] Gillison ML , Trotti AM, Harris J, Eisbruch A, Harari PM, Adelstein DJ, . Radiotherapy plus cetuximab or cisplatin in human papillomavirus-positive oropharyngeal cancer (NRG Oncology RTOG 1016): a randomised, multicentre, non-inferiority trial. Lancet 2019;393:40–50. 30449625 10.1016/S0140-6736(18)32779-X PMC6541928

[10] Chin LJ , Ratner E, Leng S, Zhai R, Nallur S, Babar I, . A SNP in a let-7 microRNA complementary site in the KRAS 3' untranslated region increases non-small cell lung cancer risk. Cancer Res 2008;68:8535–40. 18922928 10.1158/0008-5472.CAN-08-2129 PMC2672193

[11] Weidhaas J , Kim E, Herbst R, Yu J, Slack F, Blumenschein G, . The KRAS-variant and treatment response in BATTLE-1. J Clin Oncol 32:15s, 2014 (suppl; abstr 8135).

[12] Paranjape T , Heneghan H, Lindner R, Keane FK, Hoffman A, Hollestelle A, . A 3'-untranslated region KRAS variant and triple-negative breast cancer: a case-control and genetic analysis. Lancet Oncol 2011;12:377–86. 21435948 10.1016/S1470-2045(11)70044-4 PMC3488438

[13] Saridaki Z , Weidhaas J, Lenz H-J, Laurent-Puig P, Jacobs B, De Schutter J, . A let-7 microRNA-binding site polymophism is KRAS predicts improved outcome in metastatic colorectal cancer (mCRC) patients treated with salvage cetuximab/panitumumab monotherapy. Clin Cancer Res 2014;20:4499–510. 25183481 10.1158/1078-0432.CCR-14-0348 PMC4155520

[14] Chung CH , Lee JW, Slebos RJ, Howard JD, Perez J, Kang H, . A 3'-UTR KRAS-variant is associated with cisplatin resistance in patients with recurrent and/or metastatic head and neck squamous cell carcinoma. Ann Oncol 2014;25:2230–6. 25081901 10.1093/annonc/mdu367 PMC4207729

[15] Weidhaas JB , Harris J, Schaue D, Chen AM, Chin R, Axelrod R, . The *KRAS*-variant and cetuximab response in head and neck squamous cell cancer: a secondary analysis of a randomized cinical trial. JAMA Oncol 2017;3:483–91. 28006059 10.1001/jamaoncol.2016.5478 PMC5470422

[16] Yang X , Zhang X, Mortenson E, Radkevich-Brown O, Wang Y, Fu Y-X. Cetuximab-mediated tumor regression depends on innate and adaptive immune responses. Molecular Therapy 2012;21:91–100. 22990672 10.1038/mt.2012.184 PMC3538305

[17] Srivastava R , Lee S, Andrade Filho P, Lord C, Jie H, Davidson H, . Cetuximab-activated natural killer and dendritic cells collaborate to trigger tumor antigen-specific T-cell immunity in head and neck cancer patients. Clin Cancer Res 2013;19:1858–72. 23444227 10.1158/1078-0432.CCR-12-2426 PMC3640274

[18] Stephenson R , Lim C, Mattews M, Dietsch G, Hershberg R, Ferris R. TLR8 stimulation enhances cetuximab-mediated natural killer cell lysis of head and neck cancer cells and dendritic cell cross priming of EGFR-specific CD8+ T cells. Cancer Immunol Immunother 2013;62:1347–57. 23685782 10.1007/s00262-013-1437-3 PMC3720845

[19] Prentice RL , Kalbfleisch JD. Mixed discrete and continuous Cox regression model. Lifetime Data Anal 2003;9:195–210. 12735496 10.1023/a:1022935019768

[20] Bowyer S , Prithviraj P, Lorigan P, Larkin J, McArthur G, Atkinson V, . Efficacy and toxicity of treatment with the anti-CTLA-4 antibody ipilimumab in patients with metastatic melanoma after prior anti-PD-1 therapy. Br J Cancer 2016;114:1084–9. 27124339 10.1038/bjc.2016.107 PMC4865968

[21] Weidhaas JB , Eisenmann DM, Holub JM, Nallur SV. A conserved RAS/mitogen-activated protein kinase pathway regulates DNA damage-induced cell death postirradiation in Radelegans. Cancer Res 2006;66:10434–8. 17079464 10.1158/0008-5472.CAN-06-2182

[22] Weidhaas JB , Babar I, Nallur SM, Trang P, Roush S, Boehm M, . MicroRNAs as potential agents to alter resistance to cytotoxic anticancer therapy. Cancer Res 2007;67:11111–6. 18056433 10.1158/0008-5472.CAN-07-2858 PMC6070379

