# Supplementary material for: The Inherited KRAS-variant as a Biomarker of Cetuximab Response in NSCLC
Source: Cancer Res Commun. 2023 Oct 11;3(10):2074–81. doi: 10.1158/2767-9764.CRC-23-0084 (PMC10566451; doi:10.1158/2767-9764.CRC-23-0084)
Supplement: Supplementary Data Table 1 — Endpoints evaluated in RTOG 0617 [file crc-23-0084-s01.docx]

***Supplemental Table 1: Endpoints evaluated in RTOG 0617***

| *Outcome* | *Event* | *Censoring* | *Competing Risk* |
| --- | --- | --- | --- |
| **Overall survival** | Death due to any cause | Alive | NA |
| **Local failure** | The development of local progression | Alive without local progression | Dead without local progression |
| **Distant failure** | The development of distant metastasis | Alive without distant metastasis | Dead without distant metastasis |
| **Progression-free survival** | The first of: local failure, regional failure, distant failure, or death due to any cause | Alive without local, regional, or distant failure | NA |
