# Supplementary material for: The Inherited KRAS-variant as a Biomarker of Cetuximab Response in NSCLC
Source: Cancer Res Commun. 2023 Oct 11;3(10):2074–81. doi: 10.1158/2767-9764.CRC-23-0084 (PMC10566451; doi:10.1158/2767-9764.CRC-23-0084)
Supplement: Supplementary Data Table 2 — Representativeness of Study Participants [file crc-23-0084-s02.docx]

| **Supplementary Table 2.** Representativeness of Study Participants | |
| --- | --- |
| Cancer type(s)/subtype(s)/stage(s)/condition | Non-small cell lung cancer (NSCLC) |
| Considerations related to: | |
| Sex | NSCLC is slightly more common in men than women. NSCLC is divided into subtypes, the most common of which is Adenocarcinoma (40%), and then Squamous Cell (30%). |
| Age | The median age at the time of diagnoses of NSCLC is approximately 70 for men and women. |
| Race/ethnicity | In the USA in 2022, the overall NSCLC incidence rate was 61.6 cases per 100,000 in Whites and 59.5 in Blacks. The incidence was 34.3 in Asians, 62.3 in American Indian and 29.2 in Hispanic Latino, |
| Geography | Accrual was representative across the US. |
| Other considerations | Trial accrual is often less common in women and minorities. Underrepresentation in trials limits evaluation of the impact of racial/ethnic- or ancestry-based differences in efficacy and toxicity. |
| Overall representativeness of this study | The age distribution of our study is similar to the average age distribution of NSCLC nationally, with the median being 65.  The NSCLC subtype distribution was 39.1% adenocarcinoma in this study which is representative, but 45.4% squamous cell, which is higher than average.  This study was predominantly male (63%), and predominantly white (87.8%).  The study reflected the accrual patterns to trials commonly seen, yet is not completely representative of US based NSCLC populations. |
