# Supplementary material for: The Inherited KRAS-variant as a Biomarker of Cetuximab Response in NSCLC
Source: Cancer Res Commun. 2023 Oct 11;3(10):2074–81. doi: 10.1158/2767-9764.CRC-23-0084 (PMC10566451; doi:10.1158/2767-9764.CRC-23-0084)
Supplement: Supplementary Data Table 3 — KRAS Analysis Inclusion Status [file crc-23-0084-s03.docx]

| ***Supplemental Table 3: KRAS Analysis Inclusion Status*** | | | | |
| --- | --- | --- | --- | --- |
|  | **Included in KRAS Analysis (n=328)** | **Excluded: Has tissue but genotyping unsuccessful (n=5)** | **Excluded: Genotyping successful but incomplete treatment (n=7)** | **Excluded: No tissue (n=156)** |
| Treatment Arm |  |  |  |  |
| Arm A: 60 Gy | 100 (30.5%) | 3 (60.0%) | 1 (14.3%) | 48 (30.8%) |
| Arm B: 74 Gy | 68 (20.7%) | 1 (20.0%) | 0 (0.0%) | 38 (24.4%) |
| Arm C: 60 Gy + Cetuximab | 93 (28.4%) | 1 (20.0%) | 3 (42.9%) | 40 (25.6%) |
| Arm D: 74 Gy + Cetuximab | 67 (20.4%) | 0 (0.0%) | 3 (42.9%) | 30 (19.2%) |
|  | | | | |
| Assigned RT Level |  |  |  |  |
| Standard Dose: 60 Gy | 193 (58.8%) | 4 (80.0%) | 4 (57.1%) | 88 (56.4%) |
| High Dose: 74 Gy | 135 (41.2%) | 1 (20.0%) | 3 (42.9%) | 68 (43.6%) |
|  | | | | |
| Cetuximab Assignment |  |  |  |  |
| Cetuximab | 160 (48.8%) | 1 (20.0%) | 6 (85.7%) | 70 (44.9%) |
| No Cetuximab | 168 (51.2%) | 4 (80.0%) | 1 (14.3%) | 86 (55.1%) |
|  | | | | |
|  | | | | |
