# Supplementary material for: The Inherited KRAS-variant as a Biomarker of Cetuximab Response in NSCLC
Source: Cancer Res Commun. 2023 Oct 11;3(10):2074–81. doi: 10.1158/2767-9764.CRC-23-0084 (PMC10566451; doi:10.1158/2767-9764.CRC-23-0084)
Supplement: Supplementary Data Table 4 — Pretreatment Characteristics by KRAS Analysis Inclusion Status [file crc-23-0084-s04.docx]

| ***Supplemental Table 4: Pretreatment Characteristics by KRAS Analysis Inclusion Status*** | | | |
| --- | --- | --- | --- |
|  | **Included (n=328)** | **Excluded (n=168)** | **Total (n=496)** |
|  | | | |
| Age (years) |  |  |  |
| Median | 65 | 63 | 64 |
| Min - Max | 37 - 83 | 38 - 81 | 37 - 83 |
| Q1 - Q3 | 57 - 70.5 | 55.5 - 70 | 57 - 70 |
| p-value* | 0.2124 |  |  |
|  | | | |
| Gender |  |  |  |
| Male | 205 (62.5%) | 91 (54.2%) | 296 (59.7%) |
| Female | 123 (37.5%) | 77 (45.8%) | 200 (40.3%) |
| p-value* | 0.0734 |  |  |
|  | | | |
| Race |  |  |  |
| American Indian or Alaskan Native | 2 (0.6%) | 0 (0.0%) | 2 (0.4%) |
| Asian | 4 (1.2%) | 9 (5.4%) | 13 (2.6%) |
| Black or African American | 32 (9.8%) | 19 (11.3%) | 51 (10.3%) |
| Native Hawaiian or Other Pacific Islander | 1 (0.3%) | 0 (0.0%) | 1 (0.2%) |
| White | 287 (87.5%) | 137 (81.5%) | 424 (85.5%) |
| Unknown | 2 (0.6%) | 3 (1.8%) | 5 (1.0%) |
| p-value* | 0.0506 |  |  |
|  | | | |
| Ethnicity |  |  |  |
| Hispanic or Latino | 9 (2.7%) | 5 (3.0%) | 14 (2.8%) |
| Not Hispanic or Latino | 308 (93.9%) | 156 (92.9%) | 464 (93.5%) |
| Unknown | 11 (3.4%) | 7 (4.2%) | 18 (3.6%) |
| p-value* | 0.8884 |  |  |
|  | | | |
| Zubrod Performance Status |  |  |  |
| 0 | 192 (58.5%) | 93 (55.4%) | 285 (57.5%) |
| 1 | 136 (41.5%) | 75 (44.6%) | 211 (42.5%) |
| p-value* | 0.4979 |  |  |
|  | | | |
| RT Technique |  |  |  |
| 3D-CRT | 188 (57.3%) | 69 (41.1%) | 257 (51.8%) |
| IMRT | 140 (42.7%) | 99 (58.9%) | 239 (48.2%) |
| p-value* | 0.0006 |  |  |
|  | | | |
| PET Staging |  |  |  |
| No | 34 (10.4%) | 12 (7.1%) | 46 (9.3%) |
| Yes | 294 (89.6%) | 156 (92.9%) | 450 (90.7%) |
| p-value* | 0.2415 |  |  |
|  | | | |
| Histology |  |  |  |
| Squamous cell carcinoma | 148 (45.1%) | 70 (41.7%) | 218 (44.0%) |
| Adenocarcinoma | 128 (39.0%) | 67 (39.9%) | 195 (39.3%) |
| Large cell undifferentiated | 6 (1.8%) | 7 (4.2%) | 13 (2.6%) |
| Non-small cell lung cancer NOS | 46 (14.0%) | 24 (14.3%) | 70 (14.1%) |
| p-value* | 0.4499 |  |  |
|  | | | |
| Squamous cell carcinoma | 148 (45.1%) | 70 (41.7%) | 218 (44.0%) |
| Non-squamous | 180 (54.9%) | 98 (58.3%) | 278 (56.0%) |
| p-value* | 0.4631 |  |  |
|  | | | |
| AJCC Stage |  |  |  |
| N2, undetectable NSCLC primary | 5 (1.5%) | 2 (1.2%) | 7 (1.4%) |
| N3, undetectable NSCLC primary | 1 (0.3%) | 0 (0.0%) | 1 (0.2%) |
| Stage IIIA | 213 (64.9%) | 104 (61.9%) | 317 (63.9%) |
| Stage IIIB | 109 (33.2%) | 62 (36.9%) | 171 (34.5%) |
| p-value* | 0.7543 |  |  |
|  | | | |
| AJCC Stage |  |  |  |
| IIIA/N2 undetectable primary | 218 (66.5%) | 106 (63.1%) | 324 (65.3%) |
| IIIB/N3 undetectable primary | 110 (33.5%) | 62 (36.9%) | 172 (34.7%) |
| p-value* | 0.4557 |  |  |
|  | | | |
| Tumor Location |  |  |  |
| LLL or central node | 191 (58.2%) | 100 (59.5%) | 291 (58.7%) |
| Neither LLL nor central node | 137 (41.8%) | 68 (40.5%) | 205 (41.3%) |
| p-value* | 0.7821 |  |  |
|  | | | |
| RT Level |  |  |  |
| Standard Dose: 60 Gy | 193 (58.8%) | 96 (57.1%) | 289 (58.3%) |
| High Dose: 74 Gy | 135 (41.2%) | 72 (42.9%) | 207 (41.7%) |
| p-value* | 0.7166 |  |  |
|  | | | |
| RT Level (as-treated) |  |  |  |
| Standard Dose: >51 - ≤ 66 Gy | 213 (64.9%) | 88 (52.4%) | 301 (60.7%) |
| High Dose: > 66 Gy | 115 (35.1%) | 55 (32.7%) | 170 (34.3%) |
| ≤ 51 Gy | 0 (0.0%) | 25 (14.9%) | 25 (5.0%) |
| p-value* | <0.0001 |  |  |
|  | | | |
| Cetuximab |  |  |  |
| Cetuximab | 160 (48.8%) | 77 (45.8%) | 237 (47.8%) |
| No Cetuximab | 168 (51.2%) | 91 (54.2%) | 259 (52.2%) |
| p-value* | 0.5340 |  |  |
|  | | | |
| Cetuximab (as-treated) |  |  |  |
| Cetuximab | 153 (46.6%) | 68 (40.5%) | 221 (44.6%) |
| No Cetuximab | 175 (53.4%) | 100 (59.5%) | 275 (55.4%) |
| p-value* | 0.1907 |  |  |
|  | | | |
| Institution Accrual Volume |  |  |  |
| 1 patient accrued | 48 (14.6%) | 24 (14.3%) | 72 (14.5%) |
| 2-3 patients accrued | 91 (27.7%) | 32 (19.0%) | 123 (24.8%) |
| ≥ 4 patients accrued | 189 (57.6%) | 112 (66.7%) | 301 (60.7%) |
|  | | | |
| 1-3 patients accrued | 139 (42.4%) | 56 (33.3%) | 195 (39.3%) |
| ≥ 4 patients accrued | 189 (57.6%) | 112 (66.7%) | 301 (60.7%) |
| p-value* | 0.0510 |  |  |
|  | | | |
| Q1 = first quartile; Q3 = third quartile.  LLL=Lower left lobe. *p-value for age is from a t-test, all others are chi-square tests | | | |
