# Supplementary material for: The Inherited KRAS-variant as a Biomarker of Cetuximab Response in NSCLC
Source: Cancer Res Commun. 2023 Oct 11;3(10):2074–81. doi: 10.1158/2767-9764.CRC-23-0084 (PMC10566451; doi:10.1158/2767-9764.CRC-23-0084)
Supplement: Supplementary Data Table 5 — Best Observed Response by KRAS Analysis Inclusion Status [file crc-23-0084-s05.docx]

| ***Supplemental Table 5: Best Observed Response by KRAS Analysis Inclusion Status*** | | |
| --- | --- | --- |
|  | **Included (n=328)** | **Excluded (n=168)** |
|  | | |
| Complete response | 71 (21.6%) | 31 (18.5%) |
| Partial response | 133 (40.5%) | 57 (33.9%) |
| Stable disease | 52 (15.9%) | 27 (16.1%) |
| Progression | 54 (16.5%) | 30 (17.9%) |
| Not reported/evaluated | 18 (5.5%) | 23 (13.7%) |
|  | | |
| Complete/Partial response | 204 (62.2%) | 88 (52.4%) |
| All others | 124 (37.8%) | 80 (47.6%) |
| p-value* | 0.0355 |  |
|  | | |
| *p-value from a chi-square test | | |
