# Supplementary material for: The Inherited KRAS-variant as a Biomarker of Cetuximab Response in NSCLC
Source: Cancer Res Commun. 2023 Oct 11;3(10):2074–81. doi: 10.1158/2767-9764.CRC-23-0084 (PMC10566451; doi:10.1158/2767-9764.CRC-23-0084)
Supplement: Supplementary Data Table 6 — Worst Overall Treatment-Related Adverse Event by KRAS Analysis Inclusion Status [file crc-23-0084-s06.docx]

| ***Supplemental Table 6: Worst Overall Treatment-Related Adverse Event by KRAS Analysis Inclusion Status*** | | |
| --- | --- | --- |
|  | **Included (n=328)** | **Excluded (n=168)** |
|  | | |
| Worst Grade |  |  |
| None reported | 0 (0.0%) | 16 (9.5%) |
| 1 | 5 (1.5%) | 7 (4.2%) |
| 2 | 50 (15.2%) | 26 (15.5%) |
| 3 | 155 (47.3%) | 74 (44.0%) |
| 4 | 106 (32.3%) | 40 (23.8%) |
| 5 | 12 (3.7%) | 5 (3.0%) |
|  | | |
| No grade 3+ toxicity | 55 (16.8%) | 49 (29.2%) |
| Grade 3+ toxicity | 273 (83.2%) | 119 (70.8%) |
| p-value* | 0.0013 |  |
|  | | |
| *p-value from a chi-square test | | |
