# Supplementary material for: The Inherited KRAS-variant as a Biomarker of Cetuximab Response in NSCLC
Source: Cancer Res Commun. 2023 Oct 11;3(10):2074–81. doi: 10.1158/2767-9764.CRC-23-0084 (PMC10566451; doi:10.1158/2767-9764.CRC-23-0084)
Supplement: Supplementary Data Table 7 — Pretreatment Characteristics by KRAS-variant or non-variant status [file crc-23-0084-s07.docx]

| ***Supplemental Table 7: Pretreatment Characteristics by KRAS-variant or non-variant status*** | | |
| --- | --- | --- |
|  | **Wild Type (non-variant) (n=272)** | **Variant (n=56)** |
|  | | |
| Age (years) |  |  |
| Median | 65 | 64 |
| Min - Max | 38 - 83 | 37 - 82 |
| Q1 - Q3 | 58 - 70.5 | 57 - 70 |
| p-value* | 0.9259 |  |
|  | | |
| Gender |  |  |
| Male | 174 (64.0%) | 31 (55.4%) |
| Female | 98 (36.0%) | 25 (44.6%) |
| p-value* | 0.2253 |  |
|  | | |
| Race |  |  |
| American Indian or Alaskan Native | 2 (0.7%) | 0 (0.0%) |
| Asian | 4 (1.5%) | 0 (0.0%) |
| Black or African American | 30 (11.0%) | 2 (3.6%) |
| Native Hawaiian or Other Pacific Islander | 1 (0.4%) | 0 (0.0%) |
| White | 234 (86.0%) | 53 (94.6%) |
| Unknown | 1 (0.4%) | 1 (1.8%) |
| p-value* | 0.3049 |  |
|  | | |
| Ethnicity |  |  |
| Hispanic or Latino | 8 (2.9%) | 1 (1.8%) |
| Not Hispanic or Latino | 256 (94.1%) | 52 (92.9%) |
| Unknown | 8 (2.9%) | 3 (5.4%) |
| p-value* | 0.5939 |  |
|  | | |
| Zubrod Performance Status |  |  |
| 0 | 162 (59.6%) | 30 (53.6%) |
| 1 | 110 (40.4%) | 26 (46.4%) |
| p-value* | 0.4076 |  |
|  | | |
| RT Technique |  |  |
| 3D-CRT | 159 (58.5%) | 29 (51.8%) |
| IMRT | 113 (41.5%) | 27 (48.2%) |
| p-value* | 0.3581 |  |
|  | | |
| PET Staging |  |  |
| No | 24 (8.8%) | 10 (17.9%) |
| Yes | 248 (91.2%) | 46 (82.1%) |
| p-value* | 0.0434 |  |
|  | | |
| Histology |  |  |
| Squamous cell carcinoma | 127 (46.7%) | 21 (37.5%) |
| Adenocarcinoma | 103 (37.9%) | 25 (44.6%) |
| Large cell undifferentiated | 4 (1.5%) | 2 (3.6%) |
| Non-small cell lung cancer NOS | 38 (14.0%) | 8 (14.3%) |
| p-value* | 0.4682 |  |
|  | | |
| Squamous cell carcinoma | 127 (46.7%) | 21 (37.5%) |
| Non-squamous | 145 (53.3%) | 35 (62.5%) |
| p-value* | 0.2081 |  |
|  | | |
| AJCC Stage |  |  |
| N2, undetectable NSCLC primary | 5 (1.8%) | 0 (0.0%) |
| N3, undetectable NSCLC primary | 1 (0.4%) | 0 (0.0%) |
| Stage IIIA | 177 (65.1%) | 36 (64.3%) |
| Stage IIIB | 89 (32.7%) | 20 (35.7%) |
| p-value* | 0.7138 |  |
|  | | |
| AJCC Stage |  |  |
| IIIA/N2 undetectable primary | 182 (66.9%) | 36 (64.3%) |
| IIIB/N3 undetectable primary | 90 (33.1%) | 20 (35.7%) |
| p-value* | 0.7047 |  |
|  | | |
| Tumor Location |  |  |
| LLL or central node | 159 (58.5%) | 32 (57.1%) |
| Neither LLL nor central node | 113 (41.5%) | 24 (42.9%) |
| p-value* | 0.8560 |  |
|  | | |
| RT Level |  |  |
| Standard Dose: 60 Gy | 160 (58.8%) | 33 (58.9%) |
| High Dose: 74 Gy | 112 (41.2%) | 23 (41.1%) |
| p-value* | 0.9884 |  |
|  | | |
| RT Level (as-treated) |  |  |
| Standard Dose: >51 - ≤ 66 Gy | 177 (65.1%) | 36 (64.3%) |
| High Dose: > 66 Gy | 95 (34.9%) | 20 (35.7%) |
| p-value* | 0.9104 |  |
|  | | |
| Cetuximab |  |  |
| Cetuximab | 136 (50.0%) | 24 (42.9%) |
| No Cetuximab | 136 (50.0%) | 32 (57.1%) |
| p-value* | 0.3302 |  |
|  | | |
| Cetuximab (as-treated) |  |  |
| Cetuximab | 131 (48.2%) | 22 (39.3%) |
| No Cetuximab | 141 (51.8%) | 34 (60.7%) |
| p-value* | 0.2253 |  |
|  | | |
| Cetuximab Delivery |  |  |
| None delivered | 1 (0.7%) | 0 (0.0%) |
| Loading dose only | 4 (2.9%) | 2 (8.3%) |
| Concurrent cetuximab | 15 (11.0%) | 4 (16.7%) |
| Adjuvant/Consolidation cetuximab | 116 (85.3%) | 18 (75.0%) |
|  | | |
| Cetuximab Delivery |  |  |
| No adjuvant/consolidation cetuximab | 20 (14.7%) | 6 (25.0%) |
| Adjuvant/Consolidation cetuximab | 116 (85.3%) | 18 (75.0%) |
| p-value* | 0.2075 |  |
|  | | |
| Institution Accrual Volume |  |  |
| 1 patient accrued | 42 (15.4%) | 6 (10.7%) |
| 2-3 patients accrued | 74 (27.2%) | 17 (30.4%) |
| ≥ 4 patients accrued | 156 (57.4%) | 33 (58.9%) |
|  | | |
| 1-3 patients accrued | 116 (42.6%) | 23 (41.1%) |
| ≥ 4 patients accrued | 156 (57.4%) | 33 (58.9%) |
| p-value* | 0.8280 |  |
|  | | |
| Q1 = first quartile; Q3 = third quartile.  LLL=Lower left lobe. *p-value for age is from a t-test, all others are chi-square tests | | |
