# Supplementary material for: The Inherited KRAS-variant as a Biomarker of Cetuximab Response in NSCLC
Source: Cancer Res Commun. 2023 Oct 11;3(10):2074–81. doi: 10.1158/2767-9764.CRC-23-0084 (PMC10566451; doi:10.1158/2767-9764.CRC-23-0084)
Supplement: Supplementary Data Table 8 — Overall Survival [file crc-23-0084-s08.docx]

| ***Supplemental Table 8: Overall Survival*** | | | | |
| --- | --- | --- | --- | --- |
|  | **Non-variant** | | **Variant** | |
| Time (years) | % Alive (95% CI) | # at Risk | % Alive (95% CI) | # at Risk |
| 0 | 100% (N/A) | 272 | 100% (N/A) | 56 |
| 1 | 75.0% (69.4, 79.7) | 202 | 74.6% (60.9, 84.1) | 41 |
| 2 | 52.0% (45.8, 57.7) | 140 | 52.7% (38.8, 64.9) | 29 |
| 3 | 38.8% (32.9, 44.6) | 102 | 31.2% (19.3, 43.9) | 15 |
| 4 | 31.5% (26.0, 37.1) | 80 | 27.1% (15.8, 39.5) | 13 |
| 5 | 28.7% (23.4, 34.3) | 56 | 19.8% (10.0, 32.1) | 8 |
|  | | | | |
| Dead/Total | 199/272 |  | 44/56 |  |
| Median Survival Time (95% CI) | 2.1 (1.8, 2.5) |  | 2.4 (1.4, 2.5) |  |
| Hazard Ratio (95% CI) | 1.14 (0.82, 1.58) |  |  |  |
| Log rank test p-value* | 0.42 |  |  |  |
|  | | | | |
| *Two-sided log-rank, stratified by RT level (Standard dose vs. High dose) and Cetuximab (Yes vs no) | | | | |
