# Supplementary material for: The Inherited KRAS-variant as a Biomarker of Cetuximab Response in NSCLC
Source: Cancer Res Commun. 2023 Oct 11;3(10):2074–81. doi: 10.1158/2767-9764.CRC-23-0084 (PMC10566451; doi:10.1158/2767-9764.CRC-23-0084)
Supplement: Supplementary Data Table 9 — Overall Survival for non-variant and KRAS-Variant Patients by RT Level Assignment [file crc-23-0084-s09.docx]

| ***Supplemental Table 9: Overall Survival for non-variant and KRAS-Variant Patients by RT Level Assignment***  **Non-variant patients** | | | | |
| --- | --- | --- | --- | --- |
|  | **Standard Dose: >51 Gy<66 Gy** | | **High Dose: >66 Gy** | |
| Time (years) | % Alive (95% CI) | # at Risk | % Alive (95% CI) | # at Risk |
| 0 | 100% (N/A) | 177 | 100% (N/A) | 95 |
| 1 | 76.3% (69.3, 81.9) | 134 | 72.6% (62.4, 80.4) | 68 |
| 2 | 55.2% (47.6, 62.2) | 97 | 45.9% (35.6, 55.6) | 43 |
| 3 | 42.4% (35.0, 49.6) | 72 | 32.0% (22.9, 41.5) | 30 |
| 4 | 34.7% (27.7, 41.8) | 57 | 25.6% (17.3, 34.7) | 23 |
| 5 | 31.7% (24.8, 38.7) | 40 | 23.3% (15.4, 32.3) | 16 |
|  | | | | |
| Dead/Total | 125/177 |  | 74/95 |  |
| Median Survival Time (95% CI) | 2.3 (1.8, 3.0) |  | 1.9 (1.5, 2.4) |  |
| Hazard Ratio (95% CI) | 1.27 (0.95, 1.69) |  |  |  |
| p-value* | 0.13 |  |  |  |
|  | | | | |
| *Two-sided log-rank, stratified by Cetuximab (Yes vs no) | | | | |

| ***KRAS-*variant patients** | | | | |
| --- | --- | --- | --- | --- |
|  | **Standard Dose: >51 Gy<66 Gy** | | **High Dose: >66 Gy** | |
| Time (years) | % Alive (95% CI) | # at Risk | % Alive (95% CI) | # at Risk |
| 0 | 100% (N/A) | 36 | 100% (N/A) | 20 |
| 1 | 80.1% (62.7, 90.0) | 28 | 65.0% (40.3, 81.5) | 13 |
| 2 | 60.0% (42.1, 74.1) | 21 | 40.0% (19.3, 60.0) | 8 |
| 3 | 34.7% (19.1, 50.8) | 10 | 25.0% (9.1, 44.9) | 5 |
| 4 | 31.2% (16.3, 47.3) | 9 | 20.0% (6.2, 39.3) | 4 |
| 5 | 22.7% (9.6, 39.3) | 5 | 15.0% (3.7, 33.5) | 3 |
|  | | | | |
| Dead/Total | 26/36 |  | 18/20 |  |
| Median Survival Time (95% CI) | 2.5 (1.7, 3.7) |  | 1.4 (0.8, 2.4) |  |
| Hazard Ratio (95% CI) | 1.45 (0.79, 2.66) |  |  |  |
| p-value* | 0.25 |  |  |  |
|  | | | | |
| *Two-sided log-rank, stratified by Cetuximab (Yes vs no) | | | | |
|  |  |  |  |  |
