# Supplementary material for: The Inherited KRAS-variant as a Biomarker of Cetuximab Response in NSCLC
Source: Cancer Res Commun. 2023 Oct 11;3(10):2074–81. doi: 10.1158/2767-9764.CRC-23-0084 (PMC10566451; doi:10.1158/2767-9764.CRC-23-0084)
Supplement: Supplementary Data Table 10 — Overall Survival within non-variant Patients Cetuximab Assignment [file crc-23-0084-s10.docx]

| ***Supplemental Table 10: Overall Survival within non-variant Patients Cetuximab Assignment*** | | | | |  |
| --- | --- | --- | --- | --- | --- |
|  | **No Cetuximab/Loading Dose Only** | | **Cetuximab** | | |
| Time (years) | % Alive (95% CI) | # at Risk | % Alive (95% CI) | # at Risk | |
| 0 | 100% (N/A) | 141 | 100% (N/A) | 131 | |
| 1 | 73.0% (64.8, 79.5) | 102 | 77.1% (68.9, 83.4) | 100 | |
| 2 | 53.0% (44.4, 60.8) | 74 | 50.9% (42.0, 59.1) | 66 | |
| 3 | 41.3% (33.0, 49.3) | 56 | 36.1% (27.9, 44.3) | 46 | |
| 4 | 35.3% (27.4, 43.3) | 46 | 27.4% (20.0, 35.3) | 34 | |
| 5 | 32.2% (24.5, 40.1) | 32 | 25.0% (17.9, 32.7) | 24 | |
|  | | | | | |
| Dead/Total | 98/141 |  | 101/131 |  | |
| Median Survival Time (95% CI) | 2.3 (1.7, 3.0) |  | 2.0 (1.6, 2.4) |  | |
| Hazard Ratio (95% CI) | 1.18 (0.89, 1.56) |  |  |  | |
| p-value* | 0.27 |  |  |  | |
|  | | | | | |
| *Two-sided log-rank, stratified by RT level (Standard dose vs. High dose) | | | | | |
