# Supplementary material for: The Inherited KRAS-variant as a Biomarker of Cetuximab Response in NSCLC
Source: Cancer Res Commun. 2023 Oct 11;3(10):2074–81. doi: 10.1158/2767-9764.CRC-23-0084 (PMC10566451; doi:10.1158/2767-9764.CRC-23-0084)
Supplement: Supplementary Data Table 11 — Local Failure within KRAS Variant Patients by As-Treated Cetuximab [file crc-23-0084-s11.docx]

| ***Supplemental Table 11: Local Failure within KRAS Variant Patients by As-Treated Cetuximab*** | | | | |  |
| --- | --- | --- | --- | --- | --- |
|  | **No Cetuximab/Loading Dose Only** | | **Cetuximab** | | |
| Time (years) | % Alive (95% CI) | # at Risk | % Alive (95% CI) | # at Risk | |
| 0 | 0% (N/A) | 34 | 0% (N/A) | 22 | |
| 1 | 18.2% (7.2, 33.1) | 23 | 22.7% (7.9, 42.0) | 12 | |
| 2 | 33.3% (17.9, 49.6) | 13 | 36.4% (16.8, 56.4) | 9 | |
| 3 | 39.4% (22.6, 55.8) | 5 | 48.2% (23.8, 69.0) | 4 | |
| 4 | 39.4% (22.6, 55.8) | 5 | 48.2% (23.8, 69.0) | 4 | |
| 5 | 43.2% (25.0, 60.1) | 2 | 48.2% (23.8, 69.0) | 3 | |
|  | | | | | |
| Failure/Total | 15/34 |  | 10/22 |  | |
| Hazard Ratio (95% CI) | 1.05 (0.47, 2.35) |  |  |  | |
| p-value* | 0.98 |  |  |  | |
|  | | | | | |
| *Two-sided log-rank, stratified by as-treated RT level (> 51 Gy - ≤ 66 Gy vs. > 66 Gy) | | | | | |
