# Supplementary material for: The Inherited KRAS-variant as a Biomarker of Cetuximab Response in NSCLC
Source: Cancer Res Commun. 2023 Oct 11;3(10):2074–81. doi: 10.1158/2767-9764.CRC-23-0084 (PMC10566451; doi:10.1158/2767-9764.CRC-23-0084)
Supplement: Supplementary Data Table 12 — Worst Treatment-Related Toxicity By KRAS Genotype [file crc-23-0084-s12.docx]

| ***Supplemental Table 12: Worst Treatment-Related Toxicity By KRAS Genotype*** | | |
| --- | --- | --- |
|  | **Non-variant (n=272)** | **Variant (n=56)** |
| Grade 1 | 5 (1.8%) | 0 (0.0%) |
| Grade 2 | 41 (15.1%) | 9 (16.1%) |
| Grade 3 | 127 (46.7%) | 28 (50.0%) |
| Grade 4 | 90 (33.1%) | 16 (28.6%) |
| Grade 5 | 9 (3.3%) | 3 (5.4%) |
|  | | |
| No grade 3+ toxicity | 46 (16.9%) | 9 (16.1%) |
| Grade 3+ toxicity | 226 (83.1%) | 47 (83.9%) |
| p-value* | 0.6877 |  |
|  | | |
| Odds Ratio (95% CI) | 1.18 (0.53, 2.60) |  |
|  | | |
| Hematologic Toxicity |  |  |
| No grade 3+ toxicity | 112 (41.2%) | 21 (37.5%) |
| Grade 3+ toxicity | 160 (58.8%) | 35 (62.5%) |
|  | | |
| Pulmonary Toxicity |  |  |
| No grade 3+ toxicity | 216 (79.4%) | 41 (73.2%) |
| Grade 3+ toxicity | 56 (20.6%) | 15 (26.8%) |
|  | | |
| Esophagitis |  |  |
| No grade 3+ toxicity | 240 (88.2%) | 49 (87.5%) |
| Grade 3+ toxicity | 32 (11.8%) | 7 (12.5%) |
|  | | |
| *p-value is from a Cochran-Mantel-Haenzel test stratified by RT level and as-treated cetuximab assignment The logistic regression model is modeling experiencing grade 3+ toxicity and is stratified by RT level and cetuximab assignment; the referent is wild type | | |
