# Supplementary material for: The Inherited KRAS-variant as a Biomarker of Cetuximab Response in NSCLC
Source: Cancer Res Commun. 2023 Oct 11;3(10):2074–81. doi: 10.1158/2767-9764.CRC-23-0084 (PMC10566451; doi:10.1158/2767-9764.CRC-23-0084)
Supplement: Supplementary Data Table 13 — Worst Treatment-Related Toxicity Logistic Regression Model of KRAS and As-Treated RT Interaction [file crc-23-0084-s13.docx]

| ***Supplemental Table 13:*  Worst Treatment-Related Toxicity Logistic Regression Model of KRAS and As-Treated RT Interaction** | | | |
| --- | --- | --- | --- |
| Variable | Comparison | OR (95% CI) | p-value |
| KRAS mutation type | Wild type (RL) vs Variant | 0.95 (0.37, 2.41) | 0.9113 |
| RT Level | > 51 Gy - ≤ 66 Gy (RL) vs. > 66 Gy | 1.15 (0.58, 2.30) | 0.6817 |
| Interaction |  | 2.06 (0.33, 12.9) | 0.4378 |
| Model stratified by as-treated cetuximab | | | |
