# Supplementary material for: The Inherited KRAS-variant as a Biomarker of Cetuximab Response in NSCLC
Source: Cancer Res Commun. 2023 Oct 11;3(10):2074–81. doi: 10.1158/2767-9764.CRC-23-0084 (PMC10566451; doi:10.1158/2767-9764.CRC-23-0084)
Supplement: Supplementary Data Table 14 — Worst Treatment-Related Toxicity within Non-variant Patients By Cetuximab Assignment [file crc-23-0084-s14.docx]

| ***Supplemental Table 14: Worst Treatment-Related Toxicity within Non-variant Patients By Cetuximab Assignment*** | | |
| --- | --- | --- |
|  | **No Cetuximab/Loading Dose Only  (n=141)** | **Cetuximab (n=131)** |
| Grade 1 | 3 (2.1%) | 2 (1.5%) |
| Grade 2 | 30 (21.3%) | 11 (8.4%) |
| Grade 3 | 59 (41.8%) | 68 (51.9%) |
| Grade 4 | 46 (32.6%) | 44 (33.6%) |
| Grade 5 | 3 (2.1%) | 6 (4.6%) |
|  | | |
| No grade 3+ toxicity | 33 (23.4%) | 13 (9.9%) |
| Grade 3+ toxicity | 108 (76.6%) | 118 (90.1%) |
| p-value* | 0.0031 |  |
|  | | |
| Odds Ratio (95% CI) | 2.76 (1.38, 5.51) |  |
|  | | |
|  | | |
| *p-value is from a Cochran-Mantel-Haenzel test stratified by RT level assignment The logistic regression model is modeling experiencing grade 3+ toxicity and is stratified by RT level; the referent is cetuximab | | |
