# Supplementary material for: The Inherited KRAS-variant as a Biomarker of Cetuximab Response in NSCLC
Source: Cancer Res Commun. 2023 Oct 11;3(10):2074–81. doi: 10.1158/2767-9764.CRC-23-0084 (PMC10566451; doi:10.1158/2767-9764.CRC-23-0084)
Supplement: Supplementary Data Table 15 — Worst Treatment-Related Toxicity within KRAS-Variant Patients By Cetuximab [file crc-23-0084-s15.docx]

| ***Supplemental Table 15: Worst Treatment-Related Toxicity within KRAS-Variant Patients By Cetuximab*** | | |
| --- | --- | --- |
|  | **No Cetuximab/Loading Dose Only (n=34)** | **Cetuximab (n=22)** |
| Grade 2 | 9 (26.5%) | 0 (0.0%) |
| Grade 3 | 16 (47.1%) | 12 (54.5%) |
| Grade 4 | 9 (26.5%) | 7 (31.8%) |
| Grade 5 | 0 (0.0%) | 3 (13.6%) |
|  | | |
| No grade 3+ toxicity | 9 (26.5%) | 0 (0.0%) |
| Grade 3+ toxicity | 25 (73.5%) | 22 (100.0%) |
| p-value* | 0.0077 |  |
|  | | |
| Odds Ratio (95% CI) | N/A |  |
|  | | |
|  | | |
| *p-value is from a Cochran-Mantel-Haenzel test stratified by RT level assignment The logistic regression modeling could not be performed due to 0 censored patients in the cetuximab group | | |
