# Supplementary material for: The Inherited KRAS-variant as a Biomarker of Cetuximab Response in NSCLC
Source: Cancer Res Commun. 2023 Oct 11;3(10):2074–81. doi: 10.1158/2767-9764.CRC-23-0084 (PMC10566451; doi:10.1158/2767-9764.CRC-23-0084)
Supplement: Supplementary Data Table 16 — Overall Survival Squamous Cell Variant Patients Only [file crc-23-0084-s16.docx]

| ***Supplemental Table 16: Overall Survival Squamous Cell Variant Patients Only*** | | | | |
| --- | --- | --- | --- | --- |
|  | **Cetuximab** | | **No Cetuximab** | |
| Time (years) | % Alive (95% CI) | # at Risk | % Alive (95% CI) | # at Risk |
| 0 | 100% (N/A) | 13 | 100% (N/A) | 8 |
| 1 | 92.3% (56.6, 98.9) | 12 | 37.5% (8.4, 67.4) | 3 |
| 2 | 30.8% (9.5, 55.4) | 4 | 25.0% (3.7, 55.8) | 2 |
| 3 | 7.7% (0.5, 29.2) | 1 | 25.0% (3.7, 55.8) | 2 |
| 4 | 0.0% (N/A) | 0 | 25.0% (3.7, 55.8) | 2 |
| 5 | 0.0% (N/A) | 0 | 25.0% (3.7, 55.8) | 1 |
|  | | | | |
| Dead/Total | 13/13 |  | 6/8 |  |
| Median Survival Time (95% CI) | 1.5 (1.1, 2.4) |  | 0.8 (0.2, Not reached) |  |
| Hazard Ratio (95% CI) | 0.94 (0.35, 2.54) |  |  |  |
| p-value* | 0.9495 |  |  |  |
|  | | | | |
| *Two-sided log-rank, stratified by as-treated RT level (> 51 Gy - ≤ 66 Gy vs. > 66 Gy) | | | | |
